# Supplementary material for: The bactericidal FabI inhibitor Debio 1453 clears antibiotic-resistant Neisseria gonorrhoeae infection in vivo
Source: Nat Commun. 2025 Sep 18;16:8309. doi: 10.1038/s41467-025-63508-w (PMC12446476; doi:10.1038/s41467-025-63508-w)
Supplement: Supplementary file 1 — Supplemental information [file 41467_2025_63508_MOESM1_ESM.docx]

**SUPPLEMENTAL INFORMATION**

**The bactericidal FabI inhibitor Debio 1453 clears antibiotic-resistant *Neisseria gonorrhoeae* infection in vivo**

Vincent Gerusz^1,*^, Pierre Regenass^1^, Quentin Rousseau^1^, Victor Moraine^1^, Justine Dao^2^, Xavier Lavé^2^, Shampa Das^3^, Josée Hue Perron^2^, Laurence Fajas Descamps^2^, Juan Bravo^2^, Guennaëlle Dieppois^2^, Nachum Kaplan^4^, Matthew Lefebre^4^, Deanna Altomari^4^, Vladimir Romanov^4^, Terry Finn^2^, Pierre Daram^2^, Francesca Bernardini^2^, Michaël Gross^1^, Robert Lysek^1^, Aurélien Adam^1^, Danig Pohin^1^, Maurizio Maio^1^, Vasileios Tatsis^5^, Mihiro Sunose^5^, Céline Ronin^6^, Fabrice Ciesielski^6^, Josefine Ahlstrand^7^, Susanne Jacobsson^7^, Magnus Unemo^7.8^, David R. Cameron^2,*^

1. Debiopharm Research and Manufacturing SA, Martigny, Switzerland

2. Debiopharm International SA, Lausanne, Switzerland

3. Antimicrobial Pharmacodynamics and Therapeutics, Department of Pharmacology,

University of Liverpool, Liverpool Health Partners, Liverpool, United Kingdom

4. Nobelex Biotech, Inc., Toronto, Ontario, Canada

5. Sygnature Discovery, Biocity, Nottingham, United Kingdom

6. Novalix, Strasbourg, France

7. WHO Collaborating Centre for Gonorrhoea and Other STIs, National Reference Laboratory for STIs, Department of Laboratory Medicine, Faculty of Medicine and Health, Örebro University, Örebro, Sweden

8. Institute for Global Health, University College London (UCL), London, United Kingdom

*Address correspondence to:

Vincent Gerusz, Ph.D. (ORCID 0009-0005-4583-8733)

Debiopharm Research and Manufacturing SA

Rue du Levant 146. CP368

1920 Martigny, Switzerland

[vincent.gerusz@debiopharm.com](mailto:vincent.gerusz@debiopharm.com)

David R. Cameron, Ph.D. (ORCID 0000-0003-3700-5625)

Debiopharm International SA

Forum “après-demain”

Chemin Messidor 5-7.

1006 Lausanne, Switzerland

[david.cameron@debiopharm.com](mailto:david.cameron@debiopharm.com)

**Supplementary Table 1.** Elements of Structure Activity Relationships for Debio 1453 analogues and *Neisseria gonorrhoeae* FabI (*Ng*FabI).


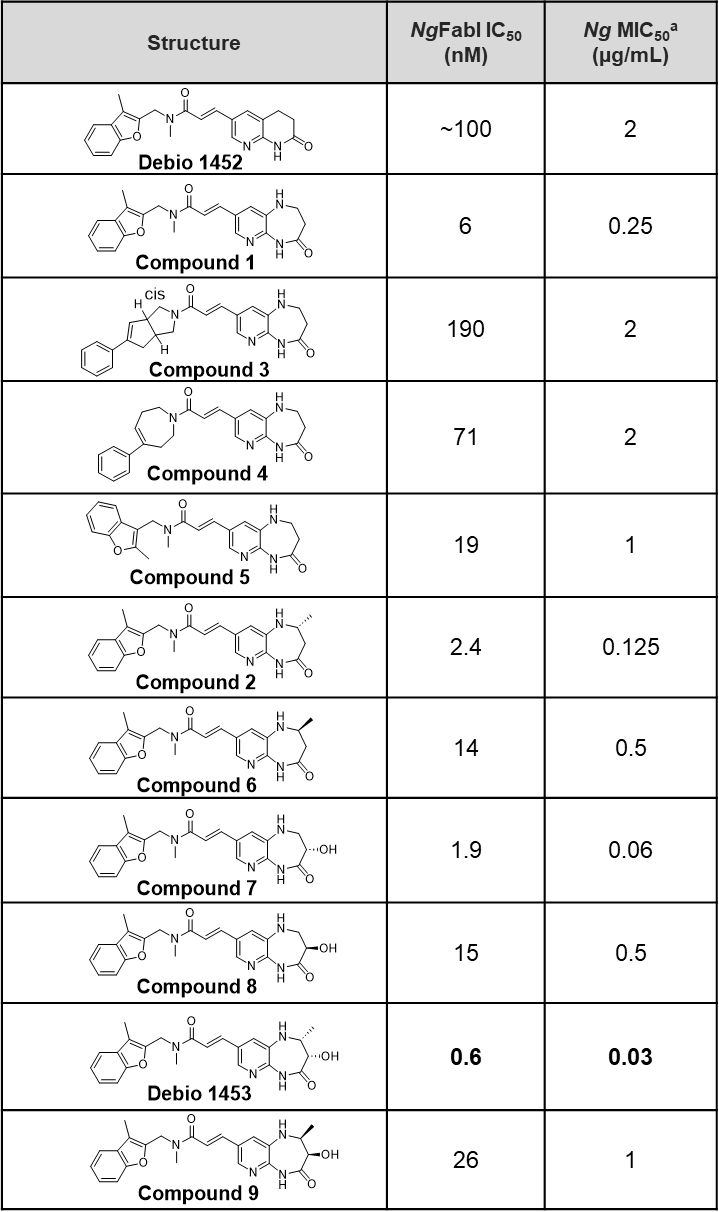


^a^Minimum inhibitory concentrations (MIC) were determined for a screening panel of 14 *N. gonorrhoeae* isolates. Source data are provided as a Source Data file.

Additional abbreviation: IC, inhibitory concentration

**Supplementary Table 2.** Ames bacterial mutation assessment for Debio 1453P.

| ***Without S9 treatment*** | | | | | | | | | | |
| --- | --- | --- | --- | --- | --- | --- | --- | --- | --- | --- |
| Strain | TA1535 | | TA1537 | | WP2 *uvrA* | | TA98 | | TA100 | |
| Debio 1453P (µg/plate) | Mean | S.E | Mean | S.E | Mean | S.E | Mean | S.E | Mean | S.E |
| 0 | 16 | 0.3 | 19 | 0.7 | 24 | 1.2 | 32 | 0.7 | 128 | 4.8 |
| 5 |  |  |  |  |  |  |  |  | 146 | 1.7 |
| 10 | 19 | 1 | 19 | 0.9 |  |  |  |  | 134 | 5.9 |
| 15.6 | 17 | 0.9 | 18 | 1.2 |  |  |  |  | 59 | 3.5 |
| 31.3 | 13 | 0.3 | 15 | 0.7 |  |  |  |  | 33 | 1.5 |
| 62.5 | 11 | 0.3 | 7 | 0.7 | 32 | 1.9 | 32 | 2 |  |  |
| 125 | 10 | 0.3 |  |  | 27 | 1 | 32 | 1.2 |  |  |
| 250 | 6 | 1.2 |  |  | 26 | 0.9 | 22 | 1.2 |  |  |
| 500 | 4 | 1 |  |  | 25 | 1.9 | 12 | 0.9 |  |  |
| 1000 |  |  |  |  | 24 | 0.3 |  |  |  |  |
| 2000 |  |  |  |  | 24 | 1.3 |  |  |  |  |
| 4000 |  |  |  |  | 22 | 1.9 |  |  |  |  |
| DMSO | 16 | 0.3 | 17 | 1.3 | 24 | 1.2 | 30 | 1.2 | 128 | 4.8 |
| Sodium azide | **433** | 16.4 |  |  |  |  |  |  | **555** | 27.1 |
| 9-Aminoacridine |  |  | **142** | 7.4 |  |  |  |  |  |  |
| MMS |  |  |  |  | **168** | 3.8 |  |  |  |  |
| 2-Nitrofluorene |  |  |  |  |  |  | **173** | 2.3 |  |  |
| ***With S9 treatment*** | | | | | | | | | | |
| Strain | TA1535 | | TA1537 | | WP2 *uvrA* | | TA98 | | TA100 | |
| Debio 1453P (µg/plate) | Mean | S.E | Mean | S.E | Mean | S.E | Mean | S.E | Mean | S.E |
| 0 | 15 | 0.9 | 21 | 1.5 | 29 | 0 | 35 | 0.9 | 134 | 0.7 |
| 5 |  |  |  |  |  |  |  |  | 141 | 0.9 |
| 10 | 18 | 1 | 17 | 1 |  |  |  |  | 128 | 2.9 |
| 15.6 | 15 | 1.3 | 18 | 0.9 |  |  |  |  | 60 | 2.1 |
| 31.3 | 16 | 0.9 | 10 | 0.6 |  |  |  |  | 30 | 0.9 |
| 62.5 | 14 | 1.2 |  |  | 32 | 1.9 | 34 | 1.9 |  |  |
| 125 | 9 | 0.7 |  |  | 30 | 0.7 | 29 | 0.9 |  |  |
| 250 | 6 | 0.7 |  |  | 31 | 0.9 | 26 | 1.5 |  |  |
| 500 | 5 | 0.3 |  |  | 34 | 1.5 | 12 | 0.9 |  |  |
| 1000 |  |  |  |  | 28 | 1.2 |  |  |  |  |
| 2000 |  |  |  |  | 26 | 0.6 |  |  |  |  |
| 4000 |  |  |  |  | 25 | 0.9 |  |  |  |  |
| DMSO | 16 | 0.3 | 24 | 1.2 | 28 | 1 | 33 | 0.3 | 129 | 3.0 |
| 2-Aminoanthracene | **104** | 6.6 | **95** | 3.5 | **187** | 4.3 | **515** | 14.4 | **1256** | 37.2 |

DMSO (50 µL/plate) served as the negative control.

Positive controls were sodium azide (1 µg/plate), 9-Aminoacridine (50 µg/plate), Methyl methanesulfonate (MMS, 500 µg/plate), 2-Nitrofluorene (2 µg/plate) and 2-Aminoanthracene (1, 2 or 20 µg/plate). Positive control values are presented in bold face for clarity.

The mean and standard error (S.E) are reported for triplicate biological measures. Source data are provided as a Source Data file.

**Supplemental Table 3. Mutation assessment using L5178Y TK +/- mouse lymphoma cells**

| **Debio 1453 (µg/mL)** | **Mutant frequency per 10^6^ cells** | **Induced mutant frequency** |
| --- | --- | --- |
| 0 | 69.3 | NA |
| 40.2 | 55.2 | NA |
| 48.2 | 58.3 | NA |
| 57.9 | 59.5 | NA |
| 69.4 | 60.1 | NA |
| 83.3 | 71.5 | 2.22 |
| 100 | 69.9 | 0.65 |
| 120 | 77 | 7.64 |
| 144 | 71.3 | 2.02 |
| MMS | **490.9** | **421.6** |

Data are the mean from two independent biological replicates, each with four technical measures. Source data are provided as a Source Data file.

NA, not applicable (frequency below untreated control)

MMS, Methyl methanesulfonate (10 µg/mL) was included as positive control, values in bold face.

**Supplementary Table 4.** In vitro micronucleus assessments in human lymphocytes for Debio 1453P

| **S9** | **Treatment time (hours)** | **Harvest time (hours)** | **Dose (µg/mL)^a^** | **Incidence of micronucleated cells (%)** | **Statistical significance^b^** |
| --- | --- | --- | --- | --- | --- |
| - | 3 | 32 | 0 | 1.53 |  |
|  |  |  | 125 | 1.13 |  |
|  |  |  | 250 | 1.20 |  |
|  |  |  | 500 | 1.43 |  |
| + | 3 | 32 | 0 | 1.15 |  |
|  |  |  | 125 | 1.95 |  |
|  |  |  | 250 | 1.16 |  |
|  |  |  | 500 | 0.70 |  |
| - | 31 | 31 | 0 | 1.38 |  |
|  |  |  | 125 | 1.00 |  |
|  |  |  | 250 | 1.23 |  |
|  |  |  | 500 | 0.93 |  |
| + | 3 | 32 | Cyclophosphamide^c^ | **3.00** | *P* = 6.50 x 10^-5^ |
| - | 31 | 31 | Colchicine^d^ | **3.35** | *P* = 3.44 x 10^-4^ |

^a^ Debio 1453P unless otherwise stated

^b^ comparisons between treatment group and the DMSO vehicle control determined using a modified chi-squared test (two-sided). Percentages are calculated from two biological measures.

^c^ 15 µg/mL

^d^ 0.08 µg/mL

Source data are provided as a Source Data file.

**Supplementary Table 5.** In vitro micronucleus assessments in human lymphocytes for Debio 1453

| **S9** | **Treatment time (hours)** | **Harvest time (hours)** | **Dose (µg/mL)^a^** | **Incidence of micronucleated cells (%)^b^** | **Statistical significance^b^** |
| --- | --- | --- | --- | --- | --- |
| - | 3 | 32.5 | 0 | 0.65 |  |
|  |  |  | 75.6 | 0.90 |  |
|  |  |  | 157 | 0.65 |  |
|  |  |  | 325 | 0.50 |  |
| + | 3 | 32.5 | 0 | 0.75 |  |
|  |  |  | 90.7 | 0.75 |  |
|  |  |  | 157 | 0.80 |  |
|  |  |  | 271 | 0.75 |  |
| - | 31 | 31 | 0 | 0.70 |  |
|  |  |  | 15.2 | 0.35 |  |
|  |  |  | 21.9 | 0.40 |  |
|  |  |  | 31.5 | 0.30 |  |
| + | 3 | 32.5 | Cyclophosphamide^c^ | **7.15** | *P* < 1.00 x 10^-7^ |
| - | 31 | 31 | Colchicine^d^ | **1.85** | *P* = 1.93 x 10^-3^ |

^a^ Debio 1453 unless otherwise stated

^b^ comparisons between treatment group and the DMSO vehicle control determined using a modified chi-squared test (two-sided). Percentages are calculated from two biological measures.

^c^ 20 µg/mL

^d^ 0.04 µg/mL

Source data are provided as a Source Data file.

**Supplementary Table 6. Cytotoxicity and cell viability assessment of Debio 1453 in HepG2 cells.** Treatments associated with cell viability < 80% and cytotoxicity > 20% are in bold font and underlined.

|  | **Cell viability (%)** | | **Cytotoxicity (%)** | |
| --- | --- | --- | --- | --- |
| **Treatment** | **Mean (n=3)** | **SD** | **Mean (n=3)** | **SD** |
| 0 µM (DMSO control) | 100 | 4 | 8 | 1 |
| **Lysis control^a^** | **1** | 0 | **100** | 4 |
| 5 µM chlorpromazine^b^ | 101 | 3 | 5 | 3 |
| **50 µM chlorpromazine** | **1** | 1 | **33** | 1 |
| **250 µM chlorpromazine** | **0** | 0 | **29** | 1 |
| 0.3 µM Debio 1453 | 106 | 6 | 7 | 0 |
| 1 µM Debio 1453 | 114 | 5 | 7 | 0 |
| 3 µM Debio 1453 | 112 | 3 | 6 | 1 |
| 10 µM Debio 1453 | 111 | 4 | 6 | 0 |
| 30 µM Debio 1453 | 114 | 7 | 6 | 0 |

^a^cells were treated with 0.5% Triton-X treatment for 60 minutes to determine maximum LDH release indicative of maximum cell lysis

^b^chlorpromazine was included as a cytotoxic positive control

DMSO, dimethyl sulfoxide; SD, standard deviation.

**Supplementary Table 7.** Data collection and refinement statistics for the *Ng*FabI-inhibitor structure.

| Complex | *Ng*FabI/NADH/Debio 1453 |
| --- | --- |
| Resolution (last shell) (Å) | 75.10-1.34 (1.44-1.34) |
| Space group | P6_1_22 |
| Unit cell | a= b= 91.26Å c=241.06Å  a= b=90° g= 120° |
| Unique reflections | 108279 |
| Completeness (last shell) (%) | 96.9 (77.7) |
| Redundancy | 19.8 |
| I/s(I) (last shell) | 26.1 (1.6) |
| Rpim (I) (last shell) (%) | 1.3 (47.2) |
| Rcryst (%) | 16.66 |
| Rfree (%) | 19.64 |
| Complex/AU | 2 |

| **Number of atoms & Average B factors (Å^2^)** | | |
| --- | --- | --- |
|  | Nb | B (av.) |
| Protein (chain A) | 1952 | 21.48 |
| Compound (chain A) | 31 | 19.30 |
| NADH (chain A) | 44 | 16.33 |
| Protein (chain B) | 1914 | 23.73 |
| Compound (chain B) | 31 | 21.25 |
| NADH (chain B) | 44 | 19.40 |
| Water molecules (chain W) | 412 | 32.09 |
| Rsmd bonds (Å) | 0.012 | |
| Rsmd angles (°) | 2.038 | |
| **Ramachandran plot** |  | |
| Residues in favored regions | 500 (97.8%) | |
| Residues in allowed regions | 11 (2.2%) | |
| Residues in outlier regions | 0 (0.0%) | |

| **A** | |
| --- | --- |
| **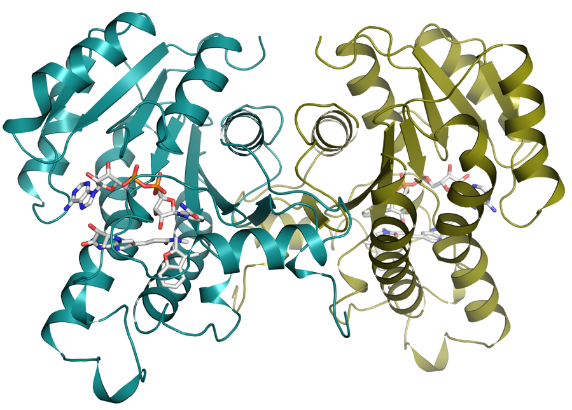** | |
| **B** | **C** |
| 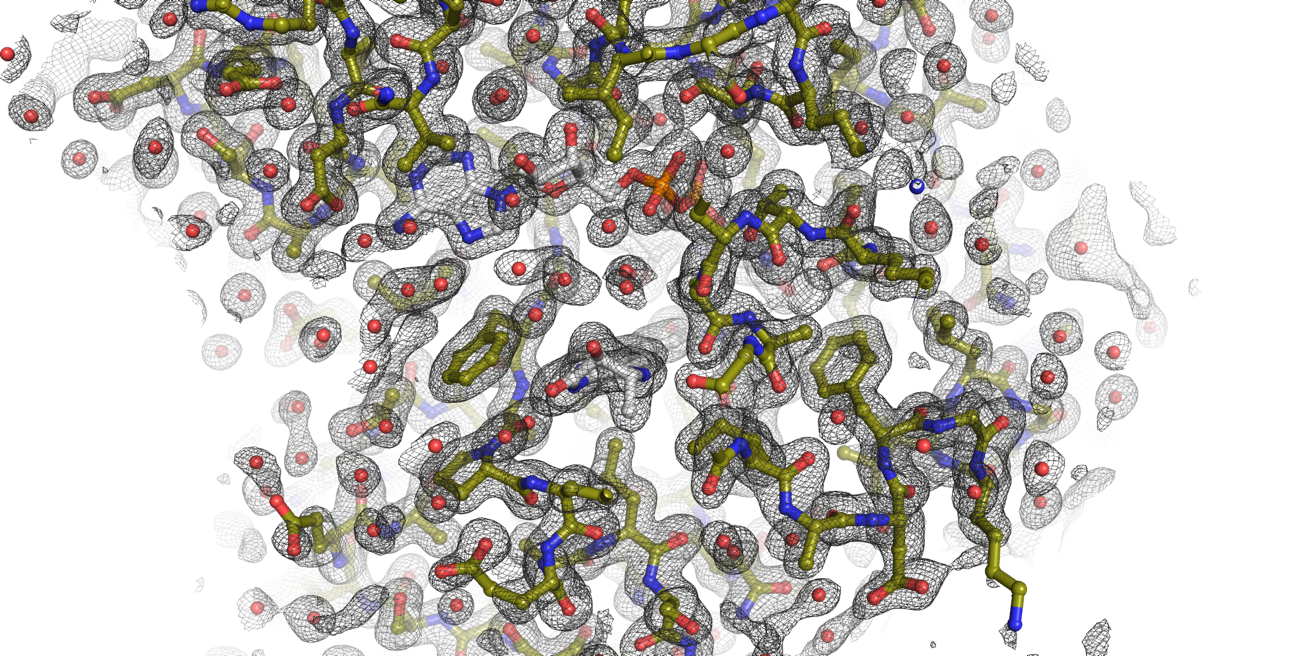 | 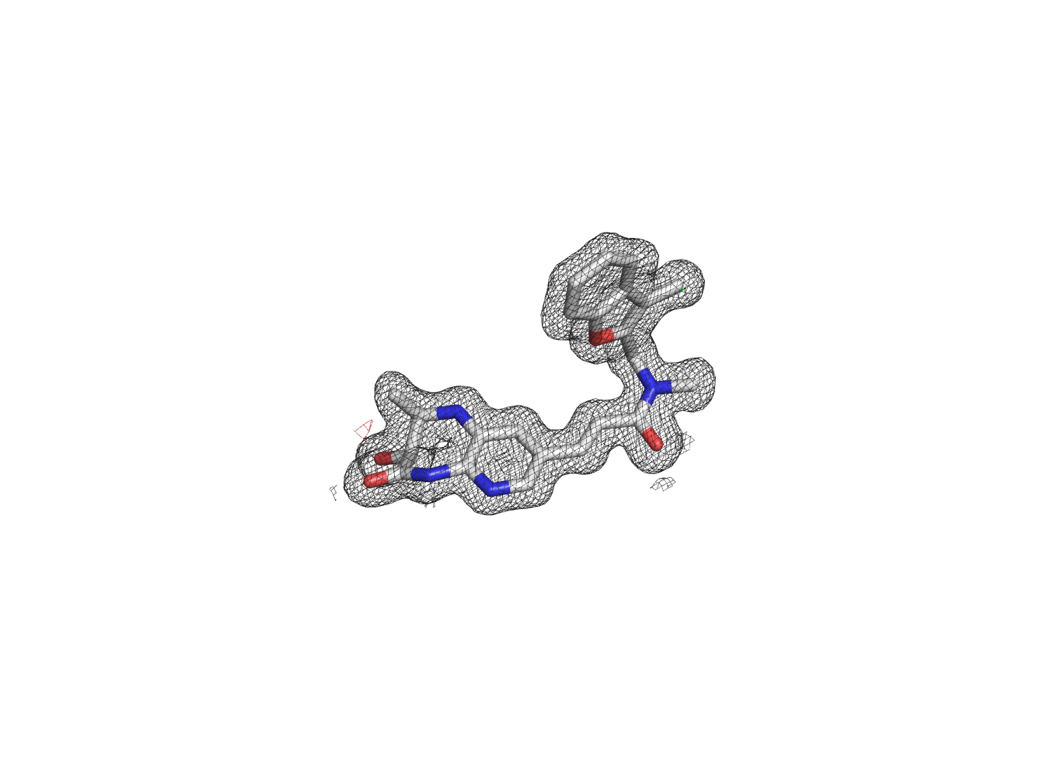 |
|  | **D** |
|  | 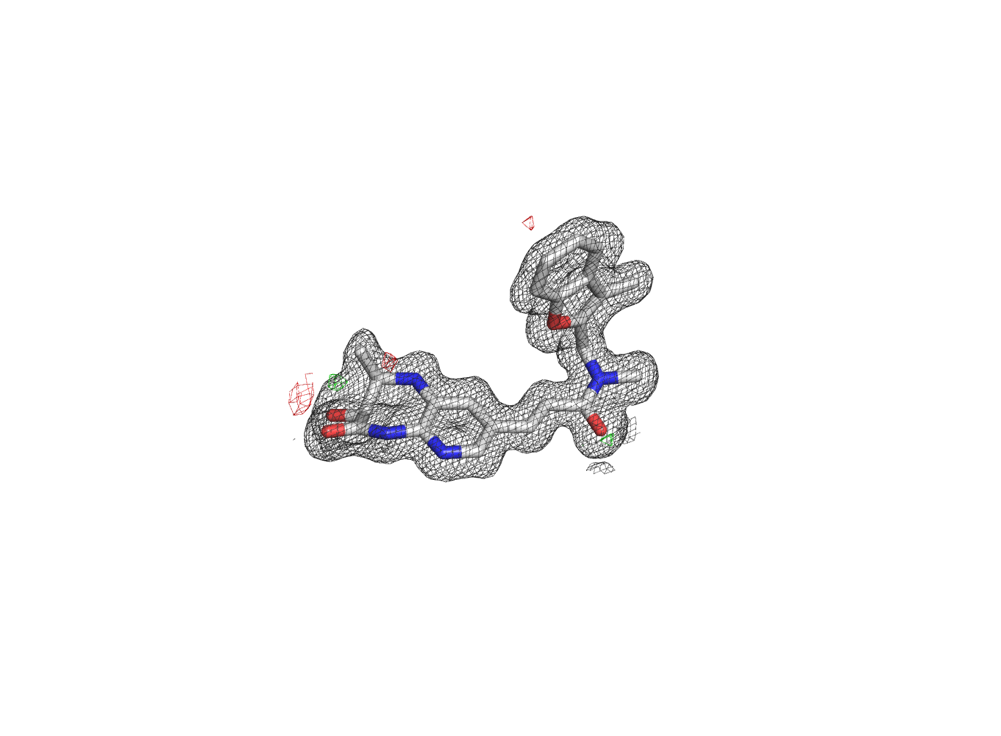 |

**Supplementary Figure 1. *Ng*FabI/NADH/Debio 1453 complex structures solved by X-Ray crystallography. (A)** Co-crystallized asymmetric unit composed of 2 *Ng*FabI/NADH/Debio 1453 complexes. FabI chains are in green and teal, while NADH cofactors and Debio 1453 are depicted as sticks. **(B)** Portion of the electron density map observed in the region of the protein active site in chain A (protein and small molecules are depicted as sticks and water molecules as red spheres – 2Fo-Fc map contoured at 0.7 σ in gray). **(C and D)** Electron density around Debio 1453 - 2Fo-Fc maps are contoured at 0.7 rmsd in gray, Fo-Fc at 3 rmsd in red (negative) and green (positive).

**
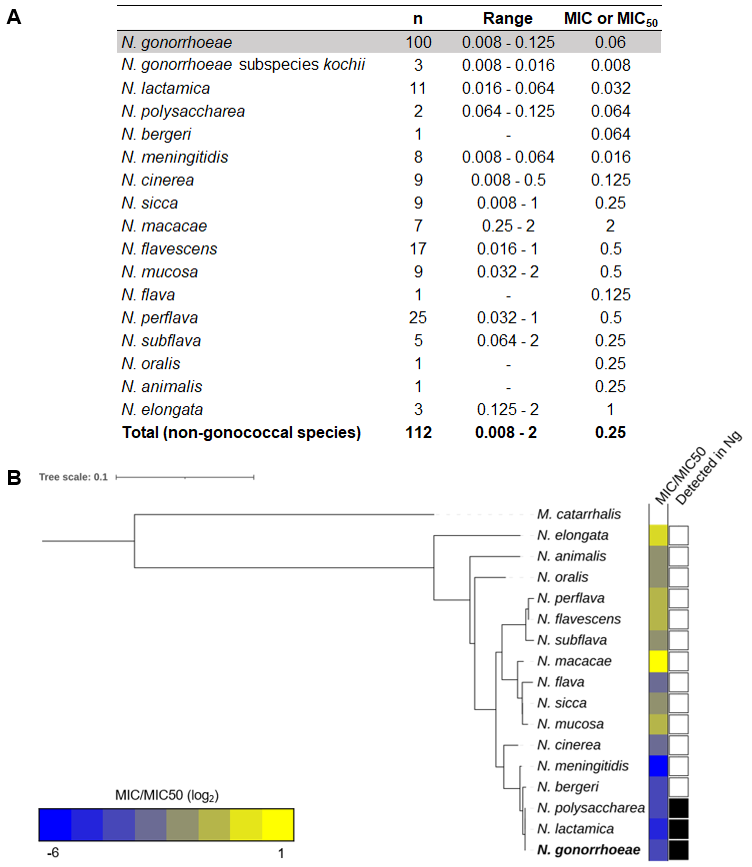
**

**Supplementary Figure 2. In vitro activity of Debio 1453 against non-gonococcal *Neisseria* species. (A)** Minimum inhibitory concentration (MIC) data for 112 isolates representing 16 different non-gonococcal *Neisseria* species. Each isolate was assessed in biological triplicate and the mode value was included in the analysis. For species where only a single isolate was tested, the MIC is presented. For species where multiple isolates were tested, the MIC inhibiting 50% of isolates (MIC_50_) is presented. **(B)** Phylogenetic relationship for FabI from each of the *Neisseria* species assessed. Coloured boxes indicate the MIC or MIC_50_ for the species. Black boxes indicate the presence of a given FabI in *N. gonorrhoeae,* white boxes indicate absence in *N. gonorrhoeae* (38,623 genomes assessed). Source data are provided as a Source Data file.

**
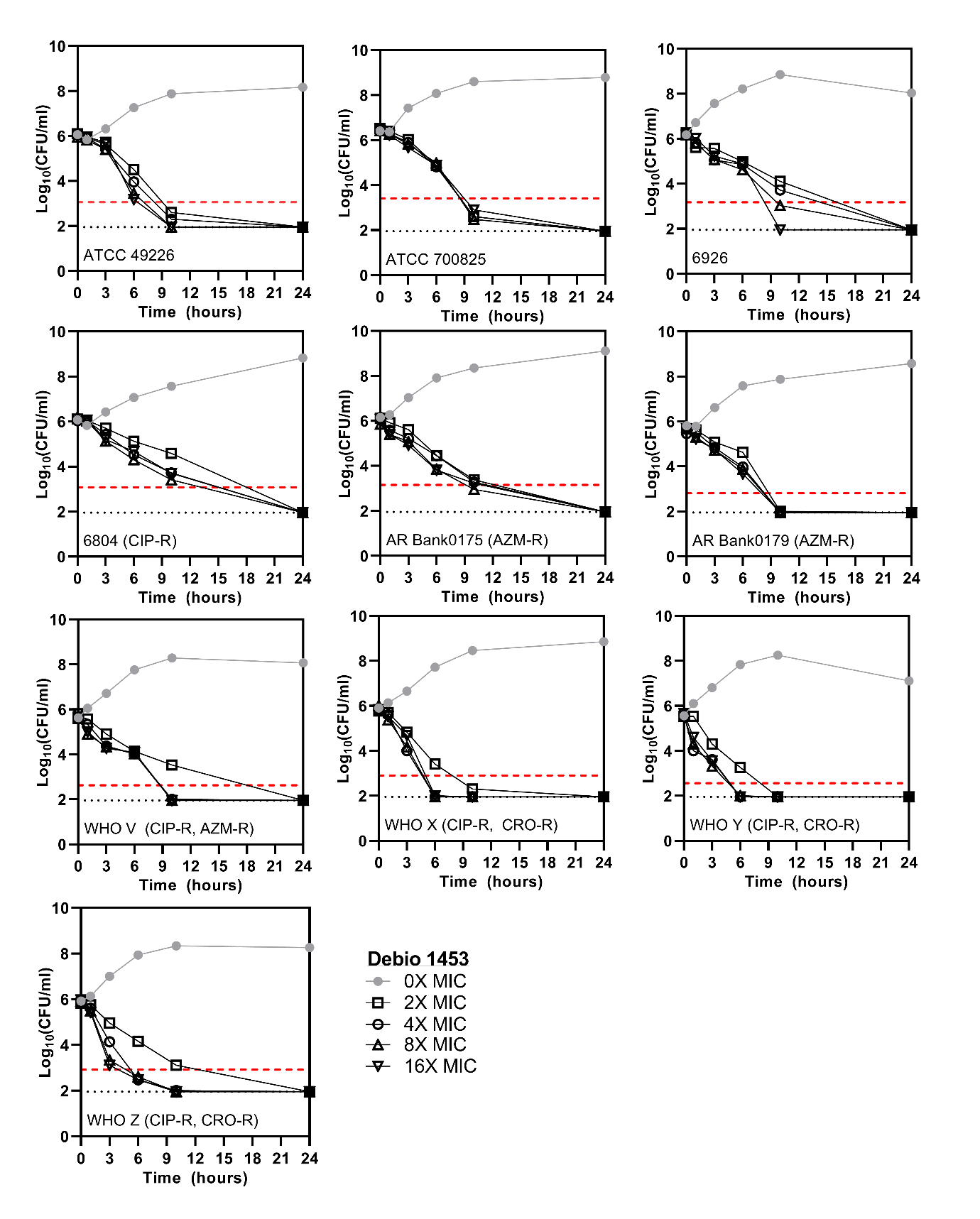
Supplementary Figure 3. Time-kill kinetics for Debio 1453 against diverse *Neisseria gonorrhoeae* isolates.** Antibiotic resistance phenotypes (resistant, R) for ciprofloxacin (CIP), azithromycin (AZM) and ceftriaxone (CRO) are provided in parenthesis. Minimum inhibitory concentrations (MIC) relevant for in vitro time-kills were determined by broth microdilution assay and were as follows: ATCC 49226, 0.125 µg/mL; ATCC 700825, 0.03 µg/mL; 6926, 0.06 µg/mL 6804, 0.06 µg/mL; AR Bank0175, 0.06 µg/mL; AR Bank0179, 0.06 µg/mL; WHO V, 0.125 µg/mL; WHO X, 0.125 µg/mL; WHO Y, 0.125 µg/mL; and WHO Z 0.125 µg/mL. Red dashed lines indicate 3log_10_ colony forming unit (CFU)/mL reductions compared to time 0. Black dotted lines are the limit of quantification. N = 1 per condition, per strain. Source data are provided as a Source Data file.


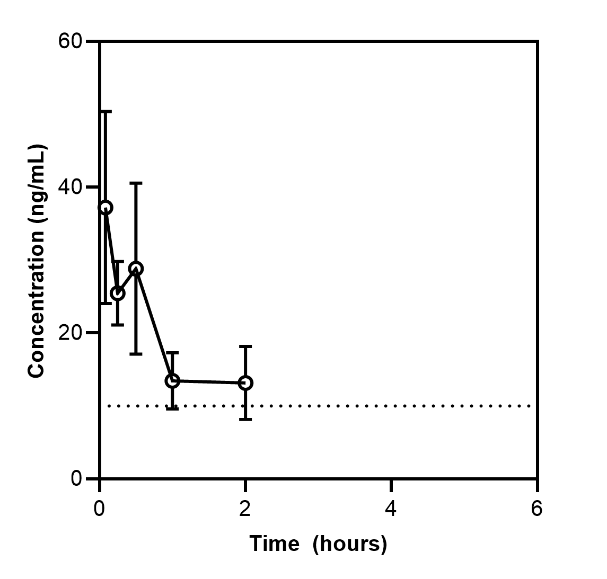


**Supplementary Figure 4. Plasma exposure of Debio 1453P in a *Neisseria gonorrhoeae* murine vaginal infection model.** Animals (n = 3 per timepoint) received a single oral dose of 80 mg/kg Debio 1453P. Debio 1453P was quantified in plasma using LC-MS/MS. The lower limit for quantification was 10 ng/mL (dotted line). Data are the mean +/- standard deviation. Source data are provided as a Source Data file.

**
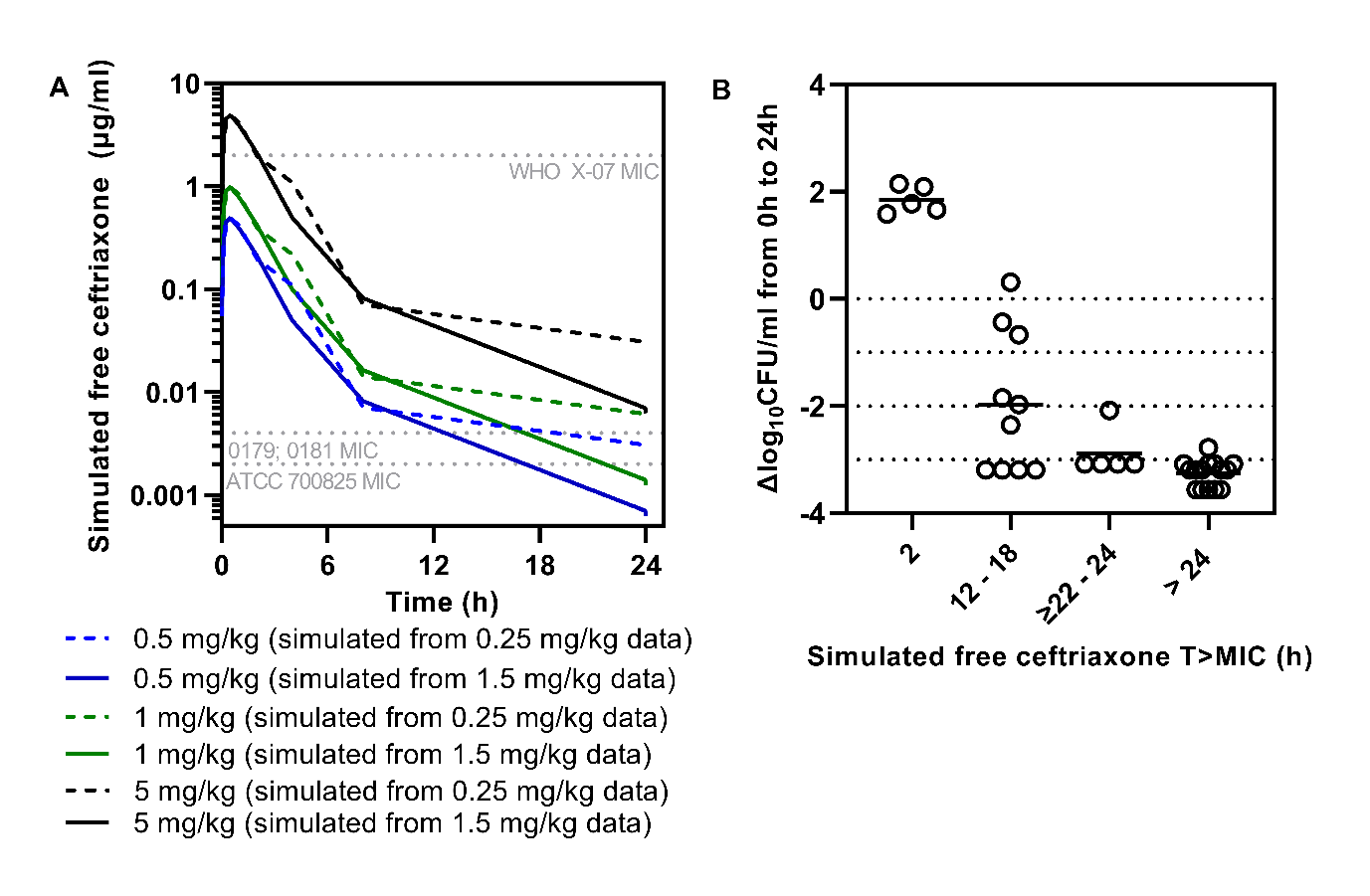
**

**Supplementary Figure 5. *Neisseria gonorrhoeae* murine vaginal infection model benchmarking with ceftriaxone. (A)** Ceftriaxone pharmacokinetic data were simulated from a previous study^1^ and related to *N. gonorrhoeae* strain minimum inhibitory concentrations (MIC; grey dotted lines) to determine the time free ceftriaxone concentrations were above MIC (*f*T>MIC). **(B)** Ceftriaxone *f*T>MIC plotted against the change in colony forming units (CFU) in vaginal lavage (n=5 animals per group). Source data are provided as a Source Data file.

**
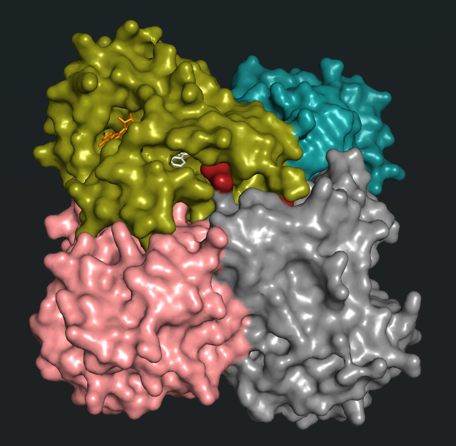
**

**Supplementary Figure 6. *Ng*FabI tetramer assembled via crystallographic symmetry from the dimer found in the asymmetric unit.** Chains A, B, A', B' are green, teal, grey and salmon, respectively with ' referring to symmetry related chains. All the chains are depicted as surface while cofactor and Debio 1453 as sticks in orange and white respectively. All Leu257 are highlighted in red. Last 2 residues (Ser 258 and Ther259) were omitted for clarity in grey subunit A’ to better display the location of its L257 interacting with subunit A.

**Supplementary Methods**

**Chemical synthesis**

*Debio 1452****.*** Prepared as described previously (referred to as compound (E)-N-methyl-N-((3-methylbenzofuran-2-yl)methyl)-3-(7-oxo-5,6,7,8-tetrahydro-1,8-naphthyridin-3-yl)acrylamide).^2^

*Compound* ***3.*** A flask was charged with (*E*)-3-(4-oxo-2,3,4,5-tetrahydro-1H-pyrido[2,3-b][1,4]diazepin-8-yl)acrylic acid trifluoroacetic acid (described previously^3^ as intermediate 143b, 49.4 mg, 145 mmol) in DMF (0.30mL). To this solution was added rel-(3aR,6aS)-5-phenyl-1,2,3,3a,4,6a-hexahydrocyclopenta[c]pyrrole 2,2,2-trifluoroacetate (described previously ^4^ as intermediate 8, 58.0 mg, 184 mmol), EDCI (51.0 mg, 253 mmol) and HOBt (33.0 mg, 239 mmol). After 2 mins of stirring at room temperature, DIPEA (88.0 µL, 488 mmol) was added and the reaction mixture was stirred at room temperature overnight. The resulting suspension was diluted with ACN, MeOH, filtered and dried under high vacuum to give the desired product as a yellow solid (35.0 mg, 56.5%). Rt 1.54 min, m/z 401.5 [M + H]^+^ (ES^+^). ^1^H NMR (DMSO-d_6_, 298K): δ, ppm 9.74 & 9.72 (rotamers, s, 1H), 7.98 & 7.96 (rotamers, d, J = 1.9 Hz, 1H), 7.49-7.46 (m, 2H), 7.37-7.22 (m, 5H), 6.87 & 6.83 (rotamers, d, J = 8.0 Hz, 1H), 6.20 (s, 1H), 6.03 & 6.00 (rotamers, t, J = 4.0 Hz, 1H), 4.03-3.75 (m, 2H), 3.72-3.48 (m, 2H), 3.45-3.35 (m, 3H), 3.19-3.09 (m, 1H), 3.03-2.90 (m, 1H), 2.66-2.55 (m, 3H).

*Compound* ***4.*** A flask was charged with (*E*)-3-(4-oxo-2,3,4,5-tetrahydro-1H-pyrido[2,3-b][1,4]diazepin-8-yl)acrylic acid trifluoroacetic acid (described previously^3^ as intermediate 143b, 93.2 mg, 239 mmol) in DMF (1.0mL). To this solution was added a mixture of the regioisomers 4-phenyl-2,3,6,7-tetrahydro-1H-azepine and 5-phenyl-2,3,4,7-tetrahydro-1H-azepine (described previously^5^ as intermediates A3a and A3b, 128 mg, 702 mmol), EDCI (75.0 mg, 372 mmol) and HOBt (52.0 mg, 377 mmol). After 2 mins of stirring at rt, DIPEA (135 µL, 766 mmol) was added and the reaction mixture was stirred at rt overnight. The resulting suspension was diluted with ACN, MeOH, H2O, filtered off and purified on PREP HPLC (0-100% ACN/water (+0.1%FA)) to give the desired product as a pale yellow solid (15.2 mg, 14.7%). Rt 2.09 min, m/z 389.5 [M + H]^+^ (ES^+^). ^1^H NMR (DMSO-d_6_, 298K): δ, ppm 9.74 (s, 1H), 8.01 (d, J = 1.9 Hz, 1H), 7.43-7.36 (m, 2H), 7.36-7.26 (m, 4H), 7.25-7.20 (m, 1H), 7.12 & 7.08 (rotamers. d, J = 15 Hz, 1H), 6.04-5.99 (m, 2H), 3.90 (t, J = 8.0 Hz, 1H), 3.82 (t, J = 8.0 Hz, 1H), 3.78 (t, J = 8.0 Hz, 1H), 3.69 (t, J = 8.0 Hz, 1H), 3.46-3.39 (m, 2H), 2.81-2.75 (m, 2H), 2.61 (t, J = 8.0 Hz, 2H), 2.50 (m, 2H) merged with DMSO signal. COSY NMR used for structure determination.

|  | Compound **4** ^1^H NMR in DMSO-d6, 278K, 400MHz | |
| --- | --- | --- |
| position | δH (multiplicity, integration) | COSY |
| 1 | 9.74 (s, 1H) | 4 |
| 2 | 6.04-5.99 (m, 2H) | 3 |
| 3 | 3.46-3.39 (m, 2H) | 2 & 4 |
| 4 | 2.61 (t, J = 8.0 Hz, 2H) | 3 & 1 |
| 5 | 7.43-7.36 (m, 2H) | 6 |
| 6 | 8.01 (d, J = 1.9 Hz, 1H) | 5 |
| 7 | 7.43-7.36 (m, 2H) | 8 |
| 8 | 7.12 & 7.08 (rotamers. d, J = 15 Hz, 1H) | 7 |
| 9 | 3.90 (t, J = 8.0 Hz, 1H) & 3.78 (t, J = 8.0 Hz, 1H) | 10 |
| 10 | 2.81-2.75 (m, 2H) | 9 |
| 11 | 6.04-5.99 (m, 2H) | 12 |
| 12 | 2.50 (m, 2H) merged with DMSO signal | 11 & 13 |
| 13 | 3.82 (t, J = 8.0 Hz, 1H) & 3.69 (t, J = 8.0 Hz, 1H) | 12 |
| 14-18 | 7.36-7.26 (m, 4H), 7.25-7.20 (m, 1H) | - |

*Compound* ***5.*** Prepared as described previously (referred to as compound 170).^6^

*Compound* ***6.*** Triethylamine (0.60 mL, 4.30 mmol) was added to a stirred solution of 5-bromo-3-fluoro-2-nitropyridine (0.30 g, 1.36 mmol) and (S)-methyl 3-aminobutanoate hydrochloride (0.21 g, 1.38 mmol) in EtOH (6.0 mL) and the reaction mixture was heated to reflux for 2 h. The reaction mixture was allowed to cool to RT, then was concentrated in vacuo and the crude material was purified by column chromatography (0-100% EtOAc/isohexane) to give (S)-Methyl 3-((5-bromo-2-nitropyridin-3-yl)amino)butanoate as a yellow solid (0.43 g, 96%).

A mixture of (S)-methyl 3-((5-bromo-2-nitropyridin-3-yl)amino)butanoate (0.43 g, 1.35 mmol), iron (0.60 g, 10.8 mmol) and NH_4_Cl (0.29 g, 5.41 mmol) in a solvent mixture of EtOH (8.0 mL) and H_2_O (2.0 mL) was stirred under reflux for ~16 hours. The reaction mixture was dry loaded on Celite^®^ and purified by column chromatography (0-100% EtOAc/isohexane) to give (S)-Methyl 3-((2-amino-5-bromopyridin-3-yl)amino)butanoate as brown solid (0.13 g, 33%). Rt 1.01 min, m/z 288/290 [M + H]^+^ (ES^+^).

Sodium hydride (60% in mineral oil, 0.03 g, 0.68 mmol) was added in small portions to a stirred solution of (S)-methyl 3-((2-amino-5-bromopyridin-3-yl)amino)butanoate (0.13 g, 0.46 mmol) in THF (5.0 mL) at 0 °C. The reaction was allowed to warm to room temperature and was stirred for 4 hours. The reaction was quenched by careful addition of saturated aqueous NH_4_Cl (50 mL) and the mixture was extracted with EtOAc (3 × 100 mL). The combined organic layers were washed with brine (1 × 100 mL), dried with MgSO_4_, concentrated in vacuo and purified by column chromatography (0-100% EtOAc/isohexane) to give (S)-8-Bromo-2-methyl-2,3-dihydro-1H-pyrido[2,3-b][1,4]diazepin-4(5H)-one as an off-white solid (50 mg, 43%). Rt 1.29 min, m/z 256/258 [M + H]^+^ (ES^+^).

A flask was charged with (S)-8-bromo-2-methyl-2,3-dihydro-1H-pyrido[2,3-b][1,4]diazepin-4(5H)-one (50.0 mg, 0.20 mmol), N-methyl-N-((3-methylbenzofuran-2-yl)methyl)acrylamide (prepared as described previously^6^ as compound 9, 45 mg, 0.20 mmol), Bu_4_NCl (6.00 mg, 0.02 mmol) and Pd-116 (10.0 mg, 0.02 mmol) and the flask was evacuated and backfilled with N_2_ three times. 1,4-Dioxane (2.5 mL) and DIPEA (0.07 mL, 0.39 mmol) were added and the reaction mixture was heated to 80 °C and stirred for 1.5 h. The solvent was removed in vacuo and the crude material was purified by column chromatography (0-5% MeOH/DCM) to give (S,E)-N-Methyl-3-(2-methyl-4-oxo-2,3,4,5-tetrahydro-1H-pyrido[2,3-b][1,4]diazepin-8-yl)-N-((3-methylbenzofuran-2-yl)methyl)acrylamide as a yellow solid (40.0 mg, 50%). Rt 1.87 min, m/z 405 [M + H]^+^ (ES^+^). ^1^H NMR (DMSO-d_6_, 363K): δ, ppm 9.31 (s, 1H), 8.01 (d, J = 1.9 Hz, 1H), 7.56 (d, J = 7.4 Hz, 1H), 7.49-7.39 (m, 3H), 7.31-7.20 (m, 2H), 7.11 (d, J = 15.7 Hz, 1H), 5.47 (s, 1H), 4.84 (s, 2H), 3.88-3.74 (m, 1H), 3.10 (s, 3H), 2.61 (dd, J = 14.4 Hz, 3.0 Hz, 1H), 2.44 (dd, J = 14.4 Hz, 7.7 Hz, 1H), 2.27 (s, 3H), 1.25 (d, J = 6.4 Hz, 3H).

*Compound* ***7.*** Prepared as described previously (referred to as compound 230).^6^

*Compound* ***8.*** Prepared as described previously (referred to as compound 229).^6^

*Compound* ***9.*** A flask was charged with *N*-methyl-*N*-((3-methylbenzofuran-2-yl)methyl)acrylamide (prepared as described previously^6^ as compound 9, 50.0 mg, 0.22 mmol), (2S,3R)-8-bromo-3-hydroxy-2-methyl-2,3-dihydro-1H-pyrido[2,3-b][1,4]diazepin-4(5H)-one (prepared as described previously^6^ as compound 148, 50.0 mg, 0.18 mmol), NBu_4_Cl (6.00 mg, 0.02 mmol) and [P(tBu)3]Pd(crotyl)Cl (Pd-162) (8.00 mg, 0.02 mmol). The vial was evacuated and backfilled with N_2_ three times, 1,4-Dioxane (3.0 mL) and N-cyclohexyl-N-methylcyclohexanamine (80.0 µL, 0.37 mmol) were added and the reaction mixture was heated to 80 °C and stirred for 3 h. The reaction was allowed to cool to RT, the solvent was removed in vacuo and the solid was washed with isohexane. The crude material was then purified by column chromatography (0-5% MeOH/DCM) and concentrated to dryness. The solid was partially dissolved in MeCN, water was added until precipitation. The precipitate was collected by filtration to give the desired product as a pale yellow solid (35.0 mg, 44%). Rt 1.83 min, m/z 421 [M + H]^+^ (ES^+^).^1^H NMR (DMSO-d_6_, 363 K): δ, ppm 9.80 (s, 1H), 7.98 (d, J = 1.9 Hz, 1H), 7.56 (d, J = 7.6 Hz, 1H), 7.49-7.38 (m, 3H), 7.32-7.21 (m, 2H), 7.12 (d, J = 16.0 Hz, 1H), 5.91 (d, J = 5.7 Hz, 1H), 4.84 (s, 2H), 4.76 (s, 1H), 4.22 (d, J = 3.3 Hz, 1H), 3.82-3.71 (m, 1H), 3.10 (s, 3H), 2.27 (s, 3H), 1.12 (d, J = 6.6 Hz, 3H). Absolute stereochemistry of compound **9** was determined from the co-structure of its enantiomer Debio 1453.

**References**

1 Connolly, K. L. et al. Pharmacokinetic Data Are Predictive of In Vivo Efficacy for Cefixime and Ceftriaxone against Susceptible and Resistant *Neisseria gonorrhoeae* Strains in the Gonorrhea Mouse Model. *Antimicrob. Agents Chemother.* **63**, e01644-18 (2019).

2 Pauls, H., & Ramnhaut, J. WO2008098374 Salts, prodrugs and polymorph of FabI inhibitors. (2008).

3 Takhi, M. et al. WO2013080222 Substituted pyridine derivatives as FabI inhibitors. (2013).

4 Guillemont, J.E.G. et al. US2018105525 Antibacterial cyclopenta[c]pyrrole substituted 3,4-dihydro-1H-[1,8]naphthyridinones. (2018).

5 Guillemont, J.E.G. et al. WO2014023815 New antibacterial compounds. (2014).

6 Gerusz, V. et al. WO2020099341 Antibiotic compounds, methods of manufacturing the same, pharmaceutical compositions containing the same and uses thereof. (2020).
