# Supplementary material for: The bactericidal FabI inhibitor Debio 1453 clears antibiotic-resistant Neisseria gonorrhoeae infection in vivo
Source: Nat Commun. 2025 Sep 18;16:8309. doi: 10.1038/s41467-025-63508-w (PMC12446476; doi:10.1038/s41467-025-63508-w)
Supplement: Supplementary file 2 — Reporting Summary [file 41467_2025_63508_MOESM2_ESM.pdf]

nature portfolio

Corresponding author(s): Vincent Gerusz
David R Cameron

Last updated by author(s): Aug 11, 2025

Reporting Summary

Nature Portfolio wishes to improve the reproducibility of the work that we publish. This form provides structure for consistency and transparency in reporting. For further information on Nature Portfolio policies, see our Editorial Policies and the Editorial Policy Checklist.

Statistics

For all statistical analyses, confirm that the following items are present in the figure legend, table legend, main text, or Methods section.

n/a	Confirmed	
☐	☒	The exact sample size (*n*) for each experimental group/condition, given as a discrete number and unit of measurement
☒	☐	A statement on whether measurements were taken from distinct samples or whether the same sample was measured repeatedly
☐	☒	The statistical test(s) used AND whether they are one- or two-sided<br>*Only common tests should be described solely by name; describe more complex techniques in the Methods section.*
☒	☐	A description of all covariates tested
☐	☒	A description of any assumptions or corrections, such as tests of normality and adjustment for multiple comparisons
☐	☒	A full description of the statistical parameters including central tendency (e.g. means) or other basic estimates (e.g. regression coefficient) AND variation (e.g. standard deviation) or associated estimates of uncertainty (e.g. confidence intervals)
☐	☒	For null hypothesis testing, the test statistic (e.g. *F*, *t*, *r*) with confidence intervals, effect sizes, degrees of freedom and *P* value noted<br>*Give P values as exact values whenever suitable.*
☒	☐	For Bayesian analysis, information on the choice of priors and Markov chain Monte Carlo settings
☒	☐	For hierarchical and complex designs, identification of the appropriate level for tests and full reporting of outcomes
☒	☐	Estimates of effect sizes (e.g. Cohen's *d*, Pearson's *r*), indicating how they were calculated

Our web collection on statistics for biologists contains articles on many of the points above.

Software and code

Policy information about availability of computer code

Data collection

-Co-crystallized ternary structures in Figure 2 and Supplementary Figures 1 & 5 as well as data in Supplementary Table 2 were collected using Proxima-2 beamline at Soleil synchrotron and processed using XDS, AutoPROC, CCP4, Phaser, Coot and Refmac.
- Data in Supplemental Figure 4A were digitized using WebPlotDigitizer (version 4.7).
-LC/MS/MS was performed using either of the following: Acquity UPLC coupled to QTRAP 6500 mass spectrometer; Nexera 30 series LC system coupled to an API-4500 mass spectrometer (Sciex) or Acquity UPLC coupled to a Thermo Q-Exactive Orbitrap mass spectrometer (Thermo)

Data analysis

-Co-crystallized ternary structures in Figure 2 were generated from the corresponding pdb files using Discovery Studio from Accelrys.
- All graph data was plotted using Graph Pad Prism versoin 9.3.1. Statistical comparisons were also performed using GraphPad Prism, as indicated in the text.
- Pharmacokinetic analysis was performed using Phoenix 64 WinNonlin (version 8.4.0.6172) using the extravascular models and the Linear Up Log Down calculation, sparse sampling method.
- Data in Supplemental Figure 4A were simulated using non-parametric superposition using Phoenix WinNonlin (version 8.3.4.295).
- LC/MS/MS data was analysed using either Thermo XCalibur (version 4.1.31.9) or Sciex Analyst software (version 1.6.2 or 1.7.2)

For manuscripts utilizing custom algorithms or software that are central to the research but not yet described in published literature, software must be made available to editors and reviewers. We strongly encourage code deposition in a community repository (e.g. GitHub). See the Nature Portfolio guidelines for submitting code & software for further information.

Data

Policy information about availability of data

All manuscripts must include a data availability statement. This statement should provide the following information, where applicable:

- Accession codes, unique identifiers, or web links for publicly available datasets
- A description of any restrictions on data availability
- For clinical datasets or third party data, please ensure that the statement adheres to our policy

All data supporting the findings of this study are available within the Source Data File. Structural data have been deposited and are available in the Protein Data Bank (PDB) under accession code 9S5X.

Research involving human participants, their data, or biological material

Policy information about studies with human participants or human data. See also policy information about sex, gender (identity/presentation), and sexual orientation and race, ethnicity and racism.

Reporting on sex and gender	Not applicable (no human subjects)
Reporting on race, ethnicity, or other socially relevant groupings	Not applicable (no human subjects)
Population characteristics	Not applicable (no human subjects)
Recruitment	Not applicable (no human subjects)
Ethics oversight	Not applicable (no human subjects)

Note that full information on the approval of the study protocol must also be provided in the manuscript.

Field-specific reporting

Please select the one below that is the best fit for your research. If you are not sure, read the appropriate sections before making your selection.

[x] Life sciences   [ ] Behavioural & social sciences   [ ] Ecological, evolutionary & environmental sciences

For a reference copy of the document with all sections, see nature.com/documents/nr-reporting-summary-flat.pdf

Life sciences study design

All studies must disclose on these points even when the disclosure is negative.

Sample size	The power of this analysis, 1-β, was set to be 0.95, and this analysis was conducted to calculate the required minimum number of animals per group when 2-log10 bacterial reductions were achieved compared to baseline control (2 h) and vehicle group (26 h).
Data exclusions	No data were excluded from any analyses.
Replication	Each experimental group for animal experiments was performed once per dose per strain (i.e. not replicated across multiple different experimental rounds). This is based on the 3R principles (reduce, reuse, refine). For the N. gonorrhoeae vaginal model mulitiple different strains were assessed (n = 4) and the results were reproducible (and correlated with the strains minimum inhibitory concentration for Debio 1453, as expected).<br>For in vitro time kill assays, results reported are n = 1 for the ten strains tested and were reproducible. These experiments were performed across multiple different days. Further, three of the ten strains were tested in a separate study and the results were reproducible.<br>All attempts at replication were successful.
Randomization	Mice were assigned by random numbers generated by Microsoft Excel to sort into random groups of 5 mice that were cohoused and received the same treatment.
Blinding	The measurement of bacterial counts was blinded. During the data collection and analysis, plates were coded so that the individual technician who counted the colonies did not know the animal number or the treatment condition.

Reporting for specific materials, systems and methods

We require information from authors about some types of materials, experimental systems and methods used in many studies. Here, indicate whether each material, system or method listed is relevant to your study. If you are not sure if a list item applies to your research, read the appropriate section before selecting a response.

Materials & experimental systems

n/a	Involved in the study	
x		Antibodies
	x	Eukaryotic cell lines
x		Palaeontology and archaeology
	x	Animals and other organisms
x		Clinical data
x		Dual use research of concern
x		Plants

Methods

n/a	Involved in the study	
x		ChIP-seq
x		Flow cytometry
x		MRI-based neuroimaging

Eukaryotic cell lines

Policy information about cell lines and Sex and Gender in Research

Cell line source(s)	Source of each cell line is specified in the text.<br>- HeLa229 cells were from European Collection of Authenticated Cell Cultures, London, UK<br>- Caco-2 intestinal epithelial cells using CacoReady-monolayers in 24-transwell plates (Readycell, Barcelona, Spain; Batch numbers 24 1059 102422 and 24 1128 072423).<br>- HepG2 cells (HB-8065) were from American Type Culture Collection<br>- Pooled human hepatocytes from 10-donor males (MX008001) and 10-donor females (FX008001) are a commercial product from BioIVT, West Sussex, UK i.e. not a "cell line"<br>- in vitro micronucleus testing used Human whole blood that was provided by Biopredic International (France) i.e. not a "cell line".
Authentication	- HeLa229 cells were from European Collection of Authenticated Cell Cultures, London, UK<br>- CacoReady-monolayers are a commercial product and authentication provided by Readycell, Barcelona, Spain (Batch numbers 24 1059 102422 and 24 1128 072423). Studies were performed and authenticated by Admescope (Finland).<br>- HepG2 cells (HB-8065) were authenticated at American Type Culture Collection. Cytotoxicity assays were performed and authenticated at Admescope (Finland).
Mycoplasma contamination	Not tested
Commonly misidentified lines<br>(See ICLAC register)	Not listed

Animals and other research organisms

Policy information about studies involving animals; ARRIVE guidelines recommended for reporting animal research, and Sex and Gender in Research

Laboratory animals	Female BALB/c mice 5 weeks of age were used. Animals were sourced from BioLASCO Taiwan Co., Ltd., an AAALAC-certified Charles River Licensee and rodent breeder.
Wild animals	None
Reporting on sex	The animals were female as it is a vaginal model of infection.
Field-collected samples	Not applicable
Ethics oversight	All animal studies were performed in accordance with the Guide of Care and Use of Laboratory Animals (National Research Council, 2011) and with approval of the Institute for Animal Care and Use Committee at Pharmacology Discovery Services Taiwan, Ltd.

Note that full information on the approval of the study protocol must also be provided in the manuscript.

Plants

Seed stocks	Not applicable
Novel plant genotypes	Not applicable
Authentication	Not applicable
